# Governance and implementation of solar-powered digital health systems in low- and middle-income countries: a mixed-methods study from Kenya and Ethiopia

**DOI:** 10.3389/fdgth.2026.1860667

**Published:** 2026-08-28

**Authors:** Ronald Danny Nyatuka, Mohammad Sakikhales, Pazion Cherinet, Md Shafiqur Rahman Jabin

**Affiliations:** 1 School of Computing and Engineering Sciences (SCES), Strathmore University, Nairobi, Kenya; 2 School of Computing and Engineering, University of West London, London, United Kingdom; 3 Orbit Health, Addis Ababa, Ethiopia; 4 Department of Medicine and Optometry, Linnaeus University, Kalmar, Sweden

**Keywords:** digital health, Ethiopia, governance, health information systems, health systems, implementation, Kenya, LMICs

## Abstract

**Background:**

Digital health systems are increasingly recognised as critical components of health system strengthening in low- and middle-income countries (LMICs). However, their effectiveness is often constrained by unreliable energy infrastructure, weak governance, and fragmented implementation approaches. Solar-powered digital health systems offer a promising solution, but their scalability depends on integrated governance and sustainable implementation models.

**Objective:**

This study aimed to develop a governance and implementation framework for scaling solar-powered digital health systems in LMICs, using empirical evidence from Kenya and Ethiopia.

**Methods:**

A mixed-methods study was conducted across selected rural and peri-urban health facilities in Kenya and Ethiopia. Data sources included facility-level infrastructure assessments, key informant interviews with health system stakeholders, and a multi-sectoral validation workshop. Quantitative data were analysed descriptively, while qualitative data were analysed using thematic analysis to indicate governance, coordination, and implementation dimensions. Findings were synthesised into a policy-oriented framework.

**Results:**

The study emerged from a hybrid governance model combining national-level policy oversight with decentralised implementation as critical for scalability. Cross-sector coordination, facilitated through a Joint Technical Steering Committee, emerged as essential for aligning health, energy, and information and communication technology (ICT) actors. A phased implementation pathway was developed, incorporating facility readiness assessment, system deployment, interoperability, and lifecycle-oriented maintenance supported by public–private partnerships and blended financing mechanisms. The findings highlight the importance of integrating energy infrastructure into digital health governance to ensure system functionality and sustainability.

**Conclusions:**

Solar-powered digital health systems can strengthen health service delivery in resource-constrained settings, but require coordinated governance, sustainable financing, and structured implementation strategies. The proposed framework advances an integrated energy–digital health governance nexus, offering a practical and conceptual pathway for scaling resilient digital health infrastructure in LMICs. Policymakers should prioritise cross-sector collaboration and system-level integration to move beyond pilot-driven approaches and achieve sustainable impact.

## Introduction

### The data–power–governance gap in digital health

Over the past decade, digital health has emerged as a central pillar of health system strengthening in low- and middle-income countries (LMICs), with investments in electronic medical records (EMRs), mobile health (mHealth), and health information systems accelerating across sub-Saharan Africa (1–5). These technologies have indicated substantial potential to improve clinical decision-making, enhance data quality, and support continuity of care. Despite widespread pilot implementation, large-scale, sustained integration of digital health systems remains limited, particularly in rural and peri-urban settings (6–8).

A critical but under-addressed constraint is the intersection of energy infrastructure, digital system design, and governance capacity. Reliable electricity remains unevenly distributed across LMIC health systems; in sub-Saharan Africa, only a minority of facilities have access to a consistent power supply (9, 10). In Kenya and Ethiopia, persistent energy and connectivity gaps continue to undermine the functionality of digital systems, resulting in fragmented data, system downtime, and reversion to paper-based processes. As highlighted in the feasibility findings from this study, health facilities frequently experience prolonged outages that render existing digital investments effectively “dormant hardware,” unable to deliver the intended clinical or administrative benefits.

While technical innovations such as solar-powered systems and offline-first digital architectures have been proposed to address infrastructure constraints, their successful deployment is not solely a technical challenge. Rather, it is fundamentally a governance problem, involving institutional coordination, financing arrangements, policy alignment, and accountability mechanisms. Existing literature on digital health in LMICs has largely focused on technological adoption and user-level barriers, with comparatively limited attention to the governance structures required to sustain integrated energy–digital health systems at scale (10–12).

### Why governance matters for scaling digital health

The failure of many digital health initiatives to transition from pilot to scale, often referred to as “pilotitis”, has been widely documented (13–16). A key driver of this phenomenon is weak institutional anchoring, where digital solutions are implemented as standalone projects rather than embedded within national health system frameworks. Fragmented governance across ministries of health, energy, and information and communication technology (ICT), combined with donor-driven implementation models, further exacerbates this challenge.

Integrating renewable energy solutions into digital health systems introduces additional governance complexity. Solar-powered digital health infrastructure sits at the intersection of multiple sectors, requiring coordinated action across health, energy, digital infrastructure, and financing domains. Without clear governance models, responsibilities for system ownership, maintenance, financing, and scale-up remain ambiguous, leading to sustainability risks (2, 13, 17, 18).

Findings from the multi-country feasibility study conducted in Kenya and Ethiopia underscore these challenges. Stakeholders consistently emphasised the need for institutional clarity, cross-sector coordination, and sustainable financing mechanisms to support long-term implementation. The absence of clearly defined governance structures emerged as a major barrier to scaling solar-powered digital health solutions, even where technical feasibility and stakeholder willingness were high.

At the same time, integrating energy and digital health systems presents a significant opportunity. Solar-powered, offline-capable platforms can enhance system resilience, support continuous service delivery, and improve data continuity in resource-constrained environments. When effectively governed, such systems can contribute to broader health system goals, including universal health coverage (UHC), improved supply chain management, and strengthened disease surveillance (13, 18).

### Positioning the framework within existing digital health implementation approaches

Existing digital health implementation frameworks provide important foundations for understanding how technologies are adopted, integrated, and sustained within health systems. These approaches commonly emphasise contextual readiness, organisational capacity, stakeholder engagement, implementation processes, and sustainability (19, 20). However, they generally treat infrastructure as one component of the broader implementation environment rather than as a foundational dependency linking energy availability, digital functionality, governance, and financing.

The framework proposed in this study builds on these established implementation perspectives but addresses a more specific implementation problem: the scale-up of digital health systems in settings where reliable electricity and connectivity cannot be assumed (21, 22). Rather than focusing only on whether a digital intervention is acceptable, feasible, or organisationally ready, the proposed framework explicitly links facility readiness, energy infrastructure, digital-system integration, cross-sector governance, financing, and lifecycle maintenance within a single implementation pathway.

This distinction is particularly important for solar-powered digital health systems because implementation extends beyond the health or ICT sectors. Energy-sector institutions, ministries of health, ICT authorities, subnational health administrations, private-sector providers, and financing actors must coordinate responsibilities for deployment, interoperability, maintenance, recurrent expenditure, and long-term system ownership (10, 23). The proposed framework therefore conceptualises solar-powered digital health as an integrated infrastructure and governance intervention, rather than solely as a digital health technology.

Accordingly, the framework should be understood as an evidence-informed, context-sensitive implementation and governance framework, rather than a replacement for established implementation theories (24, 25). Its principal contribution is to extend their application to the specific energy–digital health interface and to make cross-sector governance, energy reliability, financing, and lifecycle sustainability explicit determinants of digital health scale-up (21, 22).

### Study aim and contribution

This paper addresses a critical gap in the digital health literature by examining the governance and implementation models required to scale solar-powered digital health systems in LMICs. Drawing on empirical evidence from a mixed-methods feasibility study conducted across rural and peri-urban health facilities in Kenya and Ethiopia, as well as a multi-stakeholder validation workshop, the study develops a policy-oriented framework for scalable implementation.

Specifically, the paper aims to:

▪ Indicate governance models that support the integration of solar-powered digital health infrastructure within existing health systems;
▪ Analyse institutional coordination mechanisms required for cross-sector implementation;
▪ Propose a scalable deployment framework, including financing and maintenance models;
▪ Derive policy-relevant lessons for LMICs seeking to bridge infrastructure gaps in digital health.

The findings contribute both empirically and conceptually. Empirically, the study provides real-world insights into the operational, institutional, and policy challenges of deploying integrated energy–digital health solutions in diverse contexts. Conceptually, it advances an emerging “energy–digital health governance nexus”, highlighting the need to move beyond technology-centric approaches toward system-level governance frameworks.

By positioning governance as a central determinant of success, this paper responds to growing calls for more holistic approaches to digital health implementation in LMICs. The proposed model offers a pathway to transition from fragmented pilot projects to sustainable, scalable infrastructure systems that support resilient and equitable healthcare delivery.

## Methods

### Study design

This study used a mixed-methods policy analysis approach, integrating quantitative facility-level assessments with qualitative stakeholder evidence to examine the feasibility and governance requirements of solar-powered digital health systems. The analysis was embedded within the Bright Health feasibility study, which assessed a solar-powered, offline-capable digital health solution in rural and peri-urban health facilities (21, 22). The study comprised two phases: a baseline facility assessment and a stakeholder validation phase. The baseline phase combined structured facility assessments with key informant interviews, while the second phase used a multisectoral stakeholder workshop to validate preliminary findings and inform the development of the governance and implementation framework (13, 26, 27).

### Study setting and data sources

The study included health facilities from rural and peri-urban settings selected to reflect variation in geographic, infrastructural, and health-system contexts. In Kenya, 12 public health facilities were purposively selected across six counties: Kiambu, Machakos, Busia, Migori, Kilifi, and Kajiado, with two facilities selected from each county. The facilities included hospitals, health centres, and dispensaries representing both rural and peri-urban settings. In Ethiopia, six health centres were assessed across three regions: Sidama, Sheger City/Oromia, and Amhara/Debre Berhan.

Three complementary sources of evidence were used. First, structured and semi-structured observational assessments were undertaken at the facility level to document electricity supply and reliability, backup power, ICT infrastructure, connectivity, digital health systems, workforce capacity, financing arrangements, leadership support, training, maintenance capacity, and other implementation conditions. Second, one senior-management participant was purposively selected from each participating Kenyan facility for a key informant interview, giving a planned sample of 12 interview participants. Eligible participants included facility heads, assistant facility heads, or senior administrative staff with relevant institutional experience. Third, a stakeholder validation workshop was convened in Nairobi on February 27, 2026, bringing together more than 30 stakeholders from the health, digital health, energy, academic, development, and private-sector domains.

The key informant interviews were semi-structured and lasted approximately 45–60 min. The interview guide addressed technical feasibility, economic viability, health-system readiness, and stakeholder perspectives. Interviews were audio-recorded with participant consent and supplemented by researcher notes.

### Stakeholder validation workshop

The validation workshop was conducted in Nairobi on February 27, 2026. More than 30 stakeholders participated, representing the Ministry of Health, county governments, ICT and energy sectors, healthcare institutions, development partners, academia, and industry. The workshop was designed to validate the preliminary feasibility findings and collaboratively indicate governance, financing, and technical requirements for pilot deployment. Participants were divided into three thematic groups addressing (1) governance and institutional anchoring, (2) financing and sustainability, and (3) technical integration and deployment. Each group discussed predefined questions and produced structured recommendations, which were subsequently presented during a plenary session.

The workshop outputs included recommendations concerning institutional anchoring, cross-sector coordination, financing pathways, maintenance responsibilities, interoperability, facility-readiness screening, and indicators for progression from feasibility to pilot deployment.

### Analytical approach

Quantitative data from the facility assessments were analysed descriptively using frequencies, percentages, tables, and charts to characterise infrastructure availability, power reliability, connectivity, digital health readiness, and organisational capacity. For the Ethiopian component, feasibility was additionally assessed using a 0–10 scoring approach across technical, operational, economic, and social dimensions, with an overall weighted feasibility score. Qualitative data from key informant interviews, open-ended facility-assessment responses, and stakeholder workshop discussions were analysed using inductive thematic analysis. NVivo supported qualitative coding and the identification of recurring concepts, categories, subcategories, and overarching themes (28, 29).

Integration occurred during interpretation and synthesis. Quantitative facility-level findings were compared with qualitative accounts of infrastructure constraints, organisational readiness, financing, governance, and implementation requirements. Workshop findings were subsequently used to validate and refine the emerging themes and to translate empirical findings into a policy-oriented governance and implementation framework. This triangulation enabled convergence and divergence across the different evidence sources to be considered when developing the proposed framework (30, 31).

### Ethical considerations

Ethical approval for the Kenya component of the Bright Health feasibility study was granted by the Strathmore University Institutional Scientific and Ethical Review Committee (SU-ISERC) [Approval No. SU-ISERC3088/25]. Written informed consent was obtained from participating individuals. Research activities in Ethiopia were conducted in accordance with applicable local institutional and regulatory requirements.

## Results

### Facility-level empirical context

The facility-level assessments provided quantitative evidence of the infrastructural and organisational conditions underpinning the governance findings. Among the seven respondents contributing to the survey checklist, most facilities relied primarily on grid electricity (6/7, 86%), while only 2/7 (29%) reported high power reliability and 5/7 (71%) reported moderate reliability. Computer availability was also limited, with 5/7 facilities (71%) reporting limited availability. Similarly, 5/7 respondents (71.43%) reported limited availability of ICT or digital-health personnel. Staff confidence in ICT use was reported as high by 2/7 (29%), moderate by 4/7 (57%), and low by 1/7 (14%); ongoing digital-health training was available in 5/7 facilities (71%). These findings indicate that governance arrangements for solar-powered digital health systems must address not only energy infrastructure but also technical capacity, organisational readiness, and continuing workforce support.

This infrastructural vulnerability was also evident in facility-level accounts. One respondent from Kilifi Gede described the practical consequences of unreliable electricity:

“For the power supply, we use electricity which is not reliable due to frequent blackouts; even as we speak now, there is a blackout. Some blocks like the outpatient block have solar, while others like maternity don't have solar. At times we are even forced to do deliveries using spotlights because of blackouts.”

This account illustrates that energy insecurity is not merely an infrastructure constraint but a system-level governance and service-continuity problem. The uneven availability of solar backup across facility units also highlights the need for implementation models that prioritise critical clinical areas, establish clear responsibility for infrastructure provision and maintenance, and integrate energy resilience into digital health planning.

### Governance model for solar-powered digital health systems

#### Emergence of a hybrid governance model

Analysis of feasibility findings and stakeholder validation processes indicated a hybrid governance model as the most viable approach for implementing solar-powered digital health systems. This model combines centralised policy oversight at the national level with decentralised implementation at subnational levels, reflecting the administrative structures of both Kenya and Ethiopia.

At the national level, ministries of health play a central role in establishing policy frameworks, defining interoperability standards, and ensuring alignment with national digital health strategies. At the subnational level, counties in Kenya and regional health bureaus in Ethiopia have implementation responsibilities that include deployment, operational management, and integration into routine service delivery.

This hybrid model addresses a critical tension in LMIC health systems: the need for standardisation and coordination, alongside context-specific adaptation. Stakeholders emphasised that purely centralised models risk creating implementation bottlenecks, while fully decentralised approaches lead to fragmentation and inconsistent standards.

#### Empirical overview of facility-level infrastructure and readiness

An overview of the facility-level assessment findings is presented in Table 1. The assessment indicated substantial variation in electricity reliability, digital infrastructure, connectivity, backup power, and technical support across participating facilities. These infrastructural conditions provided an important empirical basis for interpreting the governance and implementation requirements that emerged from the qualitative and stakeholder validation components of the study.

**Table 1:** Summary of facility-level infrastructure and readiness assessment findings.

Domain	Indicator	Finding
Power infrastructure	Facilities using grid electricity as the main power source	6/7 (86%)
Power infrastructure	Facilities using a mixed power source	1/7 (14%)
Power reliability	High reliability reported	2/7 (29%)
Power reliability	Moderate reliability reported	5/7 (71%)
Digital infrastructure	Adequate computer/laptop availability	2/7 (29%)
Digital infrastructure	Limited computer/laptop availability	5/7 (71%)
Human/technical capacity	Adequate ICT/digital-health staff availability	2/7 (28.57%)
Human/technical capacity	Limited ICT/digital-health staff availability	5/7 (71.43%)
Institutional readiness	Staff confidence in ICT use: high	2/7 (29%)
Institutional readiness	Staff confidence in ICT use: moderate	4/7 (57%)
Institutional readiness	Staff confidence in ICT use: low	1/7 (14%)
Institutional readiness	Ongoing digital-health training available	5/7 (71%)
Institutional readiness	No ongoing digital-health training	2/7 (29%)

#### Institutional roles and responsibilities

The governance model delineates roles across four key institutional domains:

1. Ministries of Health (MoH): Responsible for policy leadership, regulatory oversight, and integration of solar-powered digital systems into national health strategies. The MoH also plays a key role in aligning digital health investments with the priorities of universal health coverage (UHC).
2. Energy Sector Institutions: Including ministries or agencies responsible for energy access and renewable energy deployment. These actors support infrastructure planning, standard-setting for solar systems, and alignment with national electrification strategies.
3. ICT Authorities and Digital Health Units: Responsible for ensuring interoperability with existing systems such as DHIS2 and electronic medical records (EMRs), as well as enforcing data governance standards.
4. Private Sector and Implementation Partners: Including technology providers and energy service companies (ESCOs), which play a critical role in system deployment, maintenance, and service delivery under public–private partnership (PPP) arrangements.

This multi-actor configuration reflects the inherently cross-sectoral nature of solar-powered digital health systems. The feasibility study highlighted that unclear institutional mandates and overlapping responsibilities are major barriers to implementation, reinforcing the need for explicit role definition.

#### Governance principles for scalable implementation

**Figure 1:**
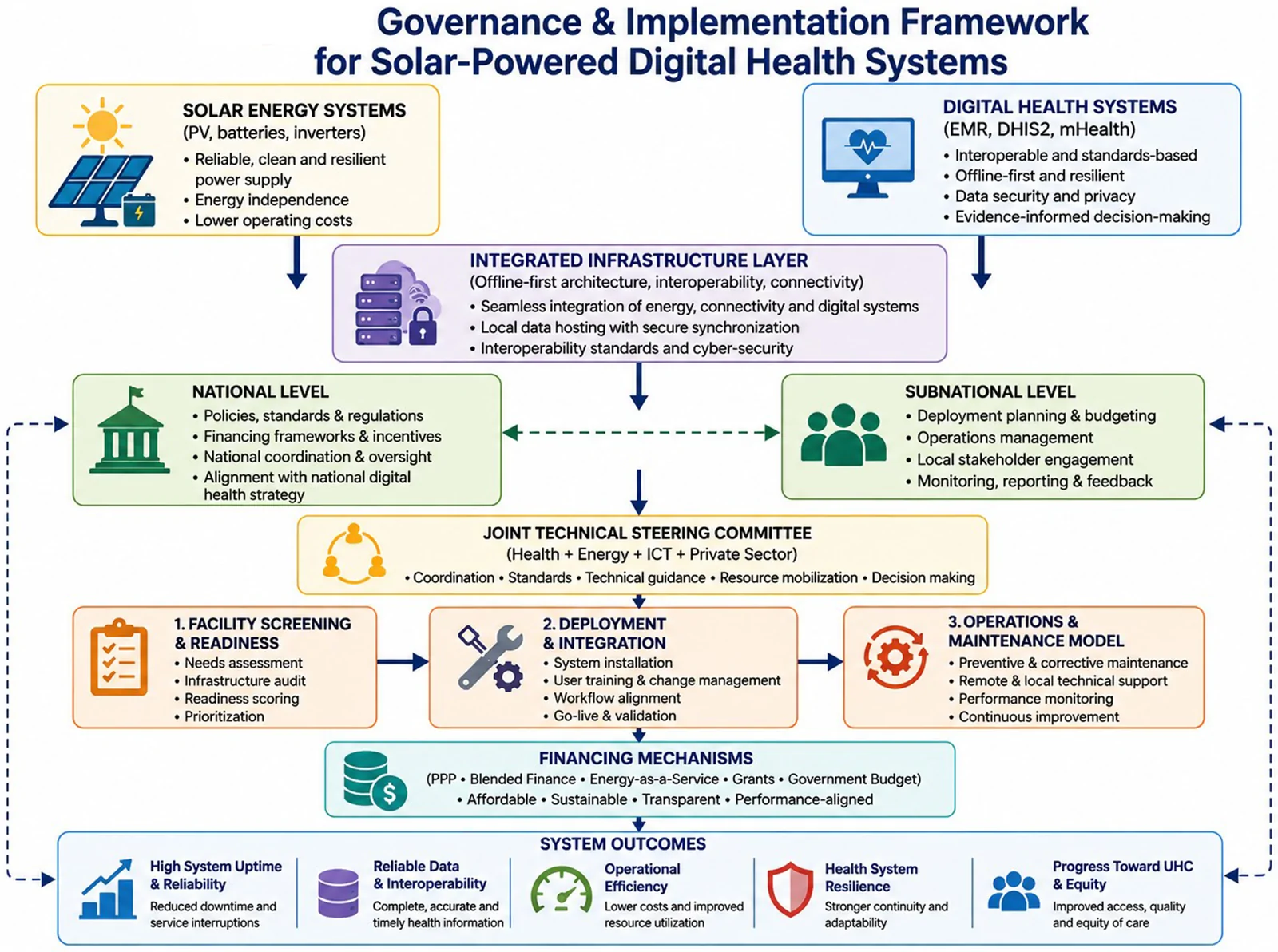
Governance and implementation framework for solar-powered digital health systems in low- and middle-income countries.

Three core governance principles emerged from the analysis:

1. Interoperability and Standardisation: Systems must align with national digital health architectures to avoid fragmentation. Interoperability emerged as a prerequisite for data continuity, system integration, and scalability.
2. Sustainability and Lifecycle Management: Governance frameworks must extend beyond initial deployment to include maintenance, financing of recurrent costs, and system upgrades. Stakeholders emphasised that many digital health projects fail due to inadequate planning for long-term sustainability.
3. Accountability and Institutional Ownership: Clear assignment of responsibility for system performance, maintenance, and financing is essential. Without defined ownership, systems risk becoming underutilised or non-functional over time. The governance and implementation framework synthesised from these findings is presented in Figure 1.

The figure illustrates how solar energy and digital health systems are integrated through an infrastructure layer within a hybrid, multi-level governance model involving national and subnational actors and a cross-sector technical steering committee. The framework outlines the implementation pathway from facility screening and deployment through maintenance and financing, with monitoring and feedback supporting system resilience, sustainability, and progress toward universal health coverage.

### Coordination and institutional architecture

#### Establishment of a joint technical steering committee

A central finding from the stakeholder validation process was the necessity of a formal cross-sector coordination mechanism, operationalised through a Joint Technical Steering Committee. This body emerged as essential for aligning actors across the health, energy, and digital sectors, which traditionally operate in silos.

The Steering Committee is envisioned as a multi-stakeholder platform comprising representatives from ministries of health, energy authorities, ICT agencies, county or regional health departments, and private sector partners. Its core functions include coordinating implementation, harmonising standards, resolving inter-institutional bottlenecks, and overseeing monitoring and evaluation processes.

“Communication with the county and budgeting for maintenance are key, and leadership willingness and proper infrastructure are necessary.” — Ngong Level 4

This cross-sector perspective was also reflected in facility-level stakeholder accounts. As one respondent noted, “Communication with the county and budgeting for maintenance are key, and leadership willingness and proper infrastructure are necessary” (Ngong Level 4).

This structure addresses a critical governance gap observed in both Kenya and Ethiopia, where fragmented institutional mandates often lead to duplication of effort, inconsistent standards, and delays in deployment. By creating a dedicated coordination platform, the model enables continuous alignment between policy, infrastructure, and implementation processes.

### Cross-sector governance and breaking institutional silos

Solar-powered digital health systems inherently span multiple sectors, requiring coordinated governance across domains that are typically managed independently. The feasibility study highlighted that the lack of coordination between health ministries, energy agencies, and ICT authorities is a major barrier to integrated implementation.

“Stakeholders should be involved in forums to discuss findings and make informed decisions, and collaboration is important to shape policies and activities.” — Ngong Level 4

The importance of coordinated stakeholder engagement was also evident in facility-level perspectives. One respondent emphasised that “Stakeholders should be involved in forums to discuss findings and make informed decisions, and collaboration is important to shape policies and activities” (Ngong Level 4).

In both Kenya and Ethiopia, stakeholders noted that digital health initiatives are often designed without consideration of energy infrastructure, while energy projects rarely account for health system requirements. This disconnect leads to suboptimal outcomes, including underutilised infrastructure and inefficient resource allocation.

The proposed governance model addresses this by institutionalising cross-sector collaboration through formal structures and shared accountability mechanisms. This aligns with broader health systems literature, which emphasises the importance of intersectoral governance in addressing complex system challenges (31, 32).

#### Multi-level governance dynamics

The implementation of solar-powered digital health systems requires coordination across multiple levels of government. In decentralised health systems such as Kenya, county governments play a critical role in service delivery, while national authorities retain responsibility for policy and standards. Similarly, in Ethiopia, regional health bureaus are central to implementation.

Findings from the feasibility study revealed both opportunities and challenges associated with this multi-level governance structure. Decentralisation enables context-specific adaptation and responsiveness to local needs. However, it also introduces variability in capacity, resource availability, and implementation approaches across regions.

**Figure 2:**
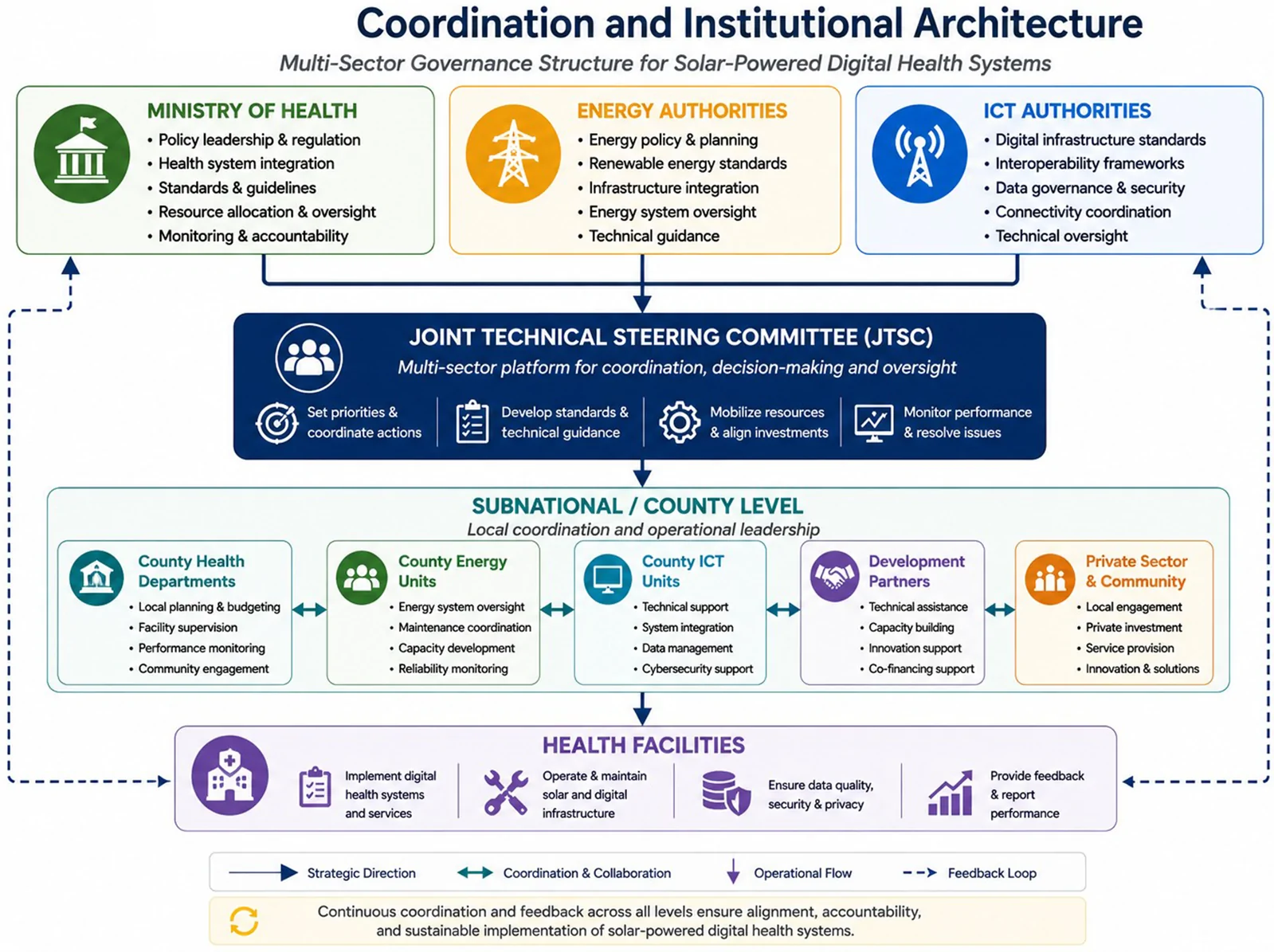
Cross-sector coordination and institutional architecture for solar-powered digital health systems in low- and middle-income countries.

The hybrid governance model mitigates these challenges by combining centralised standard-setting with decentralised execution, supported by coordination mechanisms that facilitate communication and alignment across levels. This approach reflects emerging best practices in digital health governance, which emphasise the need for flexible yet coordinated systems (2, 33). The cross-sector coordination and institutional architecture emerged through this analysis are synthesised in Figure 2.

The figure illustrates a multi-level governance structure linking national health, energy, and ICT authorities through a Joint Technical Steering Committee to subnational actors and health facilities. The committee supports cross-sector coordination, standards, resource alignment, and oversight, while subnational and facility-level actors support implementation, maintenance, and performance reporting. Feedback mechanisms connect implementation experience with higher-level policy and governance, supporting coordinated and sustainable implementation.

### Implementation and deployment framework

#### Facility readiness and screening

A critical prerequisite for successful deployment that emerged in the study is the systematic assessment of facility readiness. Stakeholders emphasised that not all facilities are equally prepared to adopt solar-powered digital health systems, necessitating a structured screening process.

Key readiness criteria include:

- Availability of basic infrastructure (space, security, hardware)
- Existing digital system usage (e.g., EMRs, DHIS2)
- Reliability of current power and connectivity
- Staff capacity and digital literacy

The feasibility findings suggested significant variability across facilities, with some exhibiting high levels of readiness while others lacked foundational infrastructure. This underscores the importance of targeted deployment strategies rather than uniform rollout approaches.

#### Standardisation and interoperability

Interoperability emerged as a central requirement for scaling solar-powered digital health systems. Systems must integrate seamlessly with existing national platforms, including DHIS2 and EMRs, to ensure data continuity and avoid fragmentation.

Stakeholders emphasised the need for adherence to national digital health standards and architectures, including data exchange protocols and reporting requirements. Failure to ensure interoperability risks creating parallel systems that undermine the health system's efficiency.

This aligns with global recommendations emphasising the importance of standards-based approaches in digital health implementation (32).

#### Maintenance and service delivery models

Long-term sustainability depends on effective maintenance models that extend beyond initial deployment. The study indicated a preference for public–private partnership (PPP)-based service models, in which private-sector partners provide ongoing technical support, system maintenance, and performance monitoring.

This approach addresses capacity constraints within public health systems, particularly in rural settings where technical expertise may be limited. It also enables performance-based service delivery, ensuring accountability for system uptime and functionality.

Stakeholders highlighted that many digital health interventions fail due to inadequate maintenance planning, reinforcing the need for lifecycle-oriented implementation strategies.

#### Financing architecture and sustainability

Financing was indicated as a critical determinant of scalability. The study findings support a blended financing model that combines public funding, donor support, and private-sector investment.

Key financing mechanisms include:

- Public–private partnerships (PPPs)
- Energy-as-a-service models
- Climate-linked financing instruments
- Government budget allocations for recurrent costs

Importantly, stakeholders emphasised the need to clearly define responsibility for ongoing costs, including maintenance, system upgrades, and energy system replacement. In the proposed model, financing responsibilities are shared between national and subnational governments, with private sector participation in service delivery.

“It depends. It is not a decision that we can make at facility level because we usually go through the Finance department at the county level, and currently we do not have funds for a high-end new project.” — Mavoko

The feasibility findings also indicated that financing decisions were not always within the authority of individual facilities. One respondent explained: “It depends. It is not a decision that we can make at facility level because we usually go through the Finance department at the county level, and currently we do not have funds for a high-end new project” (Mavoko).

**Figure 3:**
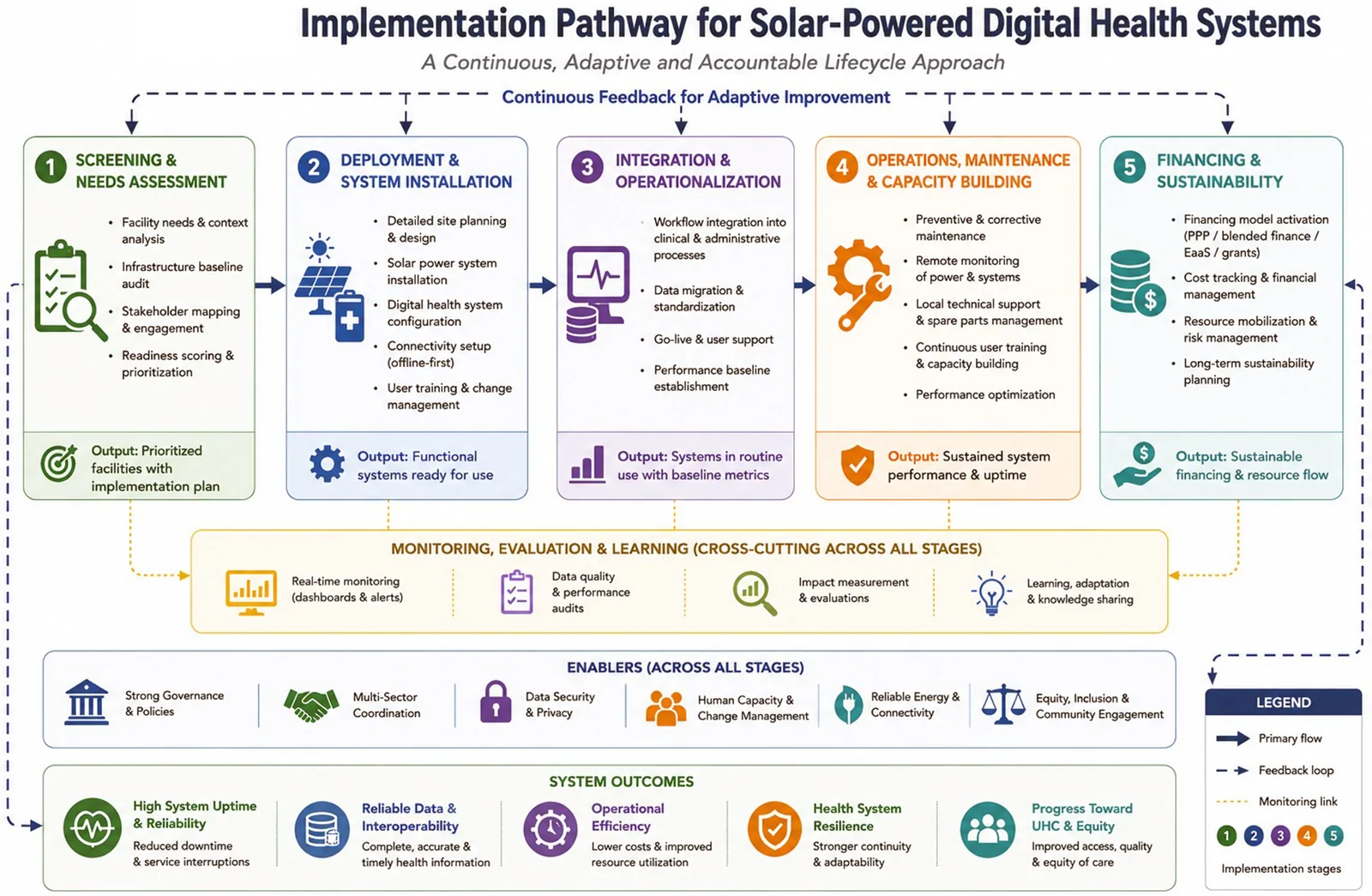
Implementation pathway for solar-powered digital health systems.

This integrated financing approach aligns with broader health financing literature, which highlights the importance of sustainable and diversified funding models in LMIC contexts (2, 13). The phased implementation and deployment pathway derived from these findings is presented in Figure 3.

The figure illustrates a phased implementation pathway from facility screening and deployment through system integration, operations and maintenance, and financing and sustainability. Cross-cutting monitoring, evaluation, learning, and enabling conditions support continuous feedback and adaptation across the implementation lifecycle. The pathway highlights the interdependence of technical, organisational, governance, and financial components in supporting system resilience, sustainability, and progress towards universal health coverage.

## Discussion

### Principal findings

This study examined the governance and implementation requirements for scaling solar-powered digital health systems in low- and middle-income countries, drawing on empirical evidence from Kenya and Ethiopia. The findings suggest that the successful deployment of such systems is not primarily a technological challenge but rather a governance and systems-integration challenge.

Three principal findings emerge from the analysis. First, a hybrid governance model that combines national-level policy oversight with decentralised implementation is essential for balancing standardisation with contextual adaptability. Second, cross-sector coordination mechanisms, particularly through a Joint Technical Steering Committee, are critical for aligning actors across the health, energy, and ICT domains and for addressing a key barrier to integrated infrastructure deployment. Third, sustainable financing and lifecycle management, including public–private partnership models and blended financing approaches, are central to ensuring long-term functionality and scalability.

These findings highlight that solar-powered digital health systems must be conceptualised as integrated infrastructure systems that require alignment across technological, institutional, and financial dimensions. The results, therefore, extend existing digital health literature by explicitly incorporating energy infrastructure into governance frameworks, an area that has received limited attention in prior research.

Existing implementation frameworks remain valuable for understanding contextual, organisational, and process-related determinants of digital health implementation. The contribution of the present framework is therefore complementary: it adds an infrastructure-governance layer for settings where energy reliability is a prerequisite for digital functionality (34–36). It explicitly connects energy and facility readiness with governance, interoperability, financing, deployment, maintenance, and institutional ownership, shifting attention from the digital intervention alone towards the wider energy–digital health infrastructure system. This distinction is particularly relevant in LMIC settings, where unreliable electricity can constrain otherwise technically appropriate digital health investments (36).

The proposed framework should be regarded as an evidence-informed framework for guiding future implementation and policy development rather than a prospectively validated model. Future studies should assess its feasibility, effectiveness, and scalability through prospective implementation and evaluation across diverse health-system contexts.

### Reframing digital health: from technology adoption to systems governance

This study highlights that the primary barrier to scaling digital health in low- and middle-income countries (LMICs) is not the lack of technology, but the absence of integrated governance systems capable of sustaining it. While solar-powered infrastructure and offline-capable digital platforms address critical technical constraints, their effectiveness ultimately depends on institutional arrangements that enable coordination, financing, and long-term system stewardship (37–40).

This finding aligns with emerging global evidence that digital health interventions often fail not because of technological limitations, but due to weak governance, fragmented implementation, and insufficient integration into health systems (12, 37, 41–44). In this context, solar-powered digital health systems represent a convergence innovation, requiring simultaneous alignment of infrastructure, policy, and service delivery systems.

By positioning governance as the central determinant of success, this study shifts the discourse from “technology deployment” to “system orchestration,” where multiple actors, sectors, and levels of governance must function in a coordinated manner.

### Governance for scale: moving beyond pilot-based models

The persistence of small-scale, donor-driven pilots—commonly referred to as “pilotitis”, remains a defining challenge in digital health (45, 46). This study helps address this challenge by proposing a governance model explicitly designed for scalability.

The hybrid governance model indicated, combining national-level policy oversight with decentralised implementation, offers a pragmatic solution to balancing standardisation with contextual adaptability. Importantly, this model is reinforced by formal coordination mechanisms, such as the Joint Technical Steering Committee, which serve to institutionalise cross-sector collaboration (17, 18, 46, 47).

The inclusion of financing and maintenance within the governance architecture is particularly significant. Many digital health initiatives fail because sustainability considerations are treated as secondary or external to implementation design. In contrast, this study integrates lifecycle financing and service delivery models as core components of governance, aligning with calls for more sustainable digital health investment strategies (17, 18, 48–50).

### Policy implications for health system strengthening in LMICs

The findings have broader implications for health system strengthening efforts in LMICs. First, institutional anchoring must be prioritised. Digital health solutions should be embedded within national health strategies and regulatory frameworks, rather than implemented as standalone projects. This ensures continuity, accountability, and alignment with broader health system goals.

Second, cross-sector governance is essential. The integration of energy and digital health systems necessitates collaboration across traditionally siloed sectors. Policymakers should establish formal coordination mechanisms that bring together health, energy, and ICT stakeholders, supported by clear mandates and shared accountability (51–54).

Third, financing models must evolve. Sustainable implementation requires moving beyond short-term project funding toward blended financing approaches that combine public investment, private sector participation, and innovative financing mechanisms such as energy-as-a-service and climate-linked funding (51, 55).

Finally, decentralisation must be supported by coordination. While decentralised governance enables local adaptation, it also introduces variability in capacity and resource allocation. National frameworks must therefore provide guidance, standards, and oversight to ensure equitable implementation across regions (51, 56–58).

### Conceptual contribution: advancing the energy–digital health governance nexus

This study advances a conceptual framework, the “energy–digital health governance nexus,” which emphasises the interdependence of infrastructure, digital systems, and governance (59, 60).

Existing digital health frameworks have largely focused on technological adoption, user engagement, and data systems (2, 12, 54). However, they often treat infrastructure and governance as external or contextual factors. This study integrates these dimensions, indicating that reliable energy, digital system functionality, and governance structures are mutually reinforcing components of a single system (59–61).

The framework contributes to the literature by:

- Positioning energy as a foundational enabler of digital health
- Highlighting governance as the integrating mechanism
- Providing a scalable model for implementation in resource-constrained settings

This integrated perspective is particularly relevant in the context of climate change and sustainability, where renewable energy solutions are increasingly linked to health system resilience (59).

### Strengths and limitations of the study

This study has several notable strengths. First, it adopts a multi-country, mixed-methods design, incorporating evidence from diverse settings in Kenya and Ethiopia. This enhances the robustness of the findings and allows for comparative insights across different governance and health system contexts. Mixed-methods approaches are particularly valuable in complex health systems research, as they enable the integration of quantitative infrastructure assessments with qualitative stakeholder perspectives, providing a more comprehensive understanding of implementation challenges (27).

Second, the study integrates empirical evidence with policy analysis, enabling the development of a framework that is both grounded in real-world data and conceptually generalisable. The inclusion of a multi-stakeholder validation workshop further strengthens the validity and applicability of the findings by incorporating perspectives from policymakers, practitioners, and technical experts. Such participatory and co-production approaches are increasingly recognised as essential for ensuring that health system interventions are contextually relevant and policy-relevant (31).

Third, the study addresses a critical but underexplored intersection between energy infrastructure and digital health systems. Explicitly linking these domains contributes to an emerging body of research highlighting the importance of integrated infrastructure in strengthening health system resilience and service delivery in resource-constrained settings. Previous studies have shown that reliable energy access is a foundational enabler of health system performance, yet it remains insufficiently integrated into digital health policy and implementation frameworks (9).

Finally, the proposed framework is action-oriented and policy-relevant, providing practical guidance for policymakers and implementers. Unlike purely theoretical models, it is grounded in feasibility findings and validated through stakeholder engagement, enhancing its applicability for real-world deployment. Implementation research emphasises the importance of such pragmatic frameworks in bridging the gap between evidence and practice, particularly in LMIC contexts (36, 62).

Despite these strengths, several limitations should be acknowledged. First, the findings are based on feasibility assessments conducted in selected regions of Kenya and Ethiopia, which may limit generalisability to other LMIC contexts. Health systems differ significantly in governance structures, institutional capacity, and resource availability, and the applicability of the proposed model may require contextual adaptation. This limitation is common in health policy and systems research, where context plays a critical role in shaping implementation outcomes (38, 63).

Second, the study focuses on early-stage feasibility and governance design, rather than long-term implementation outcomes. While the framework is informed by empirical data, its effectiveness in improving health outcomes, system efficiency, and cost-effectiveness remains to be validated through longitudinal and implementation studies. This reflects a broader gap in digital health research, where evidence on scale-up and long-term impact remains limited (2, 13).

Third, although the study incorporates perspectives from key stakeholders, it may not fully capture the views of all relevant actors, particularly frontline health workers and community-level stakeholders, who are critical to system adoption and sustainability. Previous research has highlighted the importance of user-centred design and frontline engagement in ensuring the successful implementation of digital health interventions (46).

Fourth, the analysis does not include detailed cost-effectiveness modelling, which is an important consideration for policy decision-making. While the study indicates financing models and economic considerations, further research is needed to quantify return on investment and long-term financial sustainability. Economic evaluation is a key component of health systems planning and resource allocation, particularly in LMICs with constrained budgets (64, 65).

Finally, the rapidly evolving nature of digital health and renewable energy technologies implies that governance models must remain adaptive and dynamic. The framework proposed in this study should, therefore, be viewed as a flexible model that requires continuous refinement as technologies, policies, and system contexts evolve. Adaptive governance approaches are increasingly recognised as essential for managing complexity and uncertainty in health systems (66–69).

## Conclusion

Solar-powered digital health systems represent a critical opportunity to address persistent infrastructure constraints that undermine healthcare delivery in low- and middle-income countries. However, this study suggests that their successful implementation depends not only on technological innovation but also on robust governance frameworks, coordinated institutional arrangements, and sustainable financing models.

Drawing on empirical evidence from Kenya and Ethiopia, this paper indicates a hybrid governance model that combines national-level policy oversight with decentralised implementation as a viable pathway for scaling integrated energy–digital health systems. The establishment of cross-sector coordination mechanisms, particularly a Joint Technical Steering Committee, has been shown to be essential for aligning actors across the health, energy, and digital domains. In addition, the findings highlight the importance of structured implementation processes, including facility readiness assessment, standardisation, and lifecycle-oriented maintenance models supported by blended financing approaches.

Conceptually, this study advances the notion of an energy–digital health governance nexus, underscoring the interdependence of infrastructure, technology, and institutional capacity in shaping health system performance. By shifting the focus from isolated digital interventions to integrated system design, the proposed framework offers a more holistic approach to digital health scale-up in resource-constrained settings.

For policymakers, the findings emphasise the need to move beyond pilot-driven approaches toward institutionally anchored, system-wide strategies that address both infrastructural and governance challenges. Future research should focus on evaluating the long-term impact, cost-effectiveness, and scalability of such models across diverse contexts. Ultimately, integrated governance of energy and digital health systems will be essential for advancing resilient, equitable, and sustainable healthcare delivery, and for achieving universal health coverage.

## Data Availability

The original contributions presented in the study are included in the article/Supplementary Material, further inquiries can be directed to the corresponding author.
